# Typography and Eye Weariness

**Published:** 1898-07-02

**Authors:** 


### TYPOGRAPHY AND EYE WEARINESS.

Many researches have been made as to the cause of eye fatigue in reading, and recently Messrs. Griffling and Franz, of Columbia University, have come to the conclusion that inferior size of the type is more efficient than any other cause in producing the eye weariness which so often accompanies the act of reading. They also maintain that the amount of daylight (unless it be reduced below the limits of distinct vision), and the variation, spacing, and style of type are of less importance in this relation than is the size of the type. We have no doubt that they are mainly right in their contention. It must not, however, be imagined that the difficulty entirely arises from "strain" of accommodation. The smaller the type the greater the tendency to bring the eyes nearer to the work so as to increase the size of the image, and thus the greater the strain upon the ciliary muscle. Again, the nearer the page is held the greater the convergence of the eyes necessary for the production of single vision, and thus the greater the work thrown upon the converging muscles, and the consequent strain upon them also. So far, we are dealing with elementary principles which apply to all sorts of fine work.

In reading, however, we have to do with a much more complex problem, for so soon as the eyes reach the end of a line they must spring back in an instant to the beginning of the next, a feat which involves a very complicated muscular operation, and anything which embarrasses this operation undoubtedly becomes a cause of eye weariness in reading. In this way, also, undue smallness of type operates injuriously, for, besides being more difficult to read, it is more difficult to "catch up" at the beginning of the next line. This, however, is not all, for directly we are tempted by the smallness of the type to bring the eyes nearer to the page the angle through which the eye must move to swing back from end to end of the reading line is considerably increased, as also is the muscular effort. Mere muscular effort, however, is of far less importance than the nervous strain involved in solving what the physiologists would call the "dilemma" as to which line to aim at, and this is much increased by any enlargement of the angle through which the eye has to spring back, or, what has the same effect, by any lengthening of the line. The length of line is a matter of great importance.

From the centre of distinct vision in the line of sight the acuity of vision in the different parts of the field rapidly falls away on every side, so that all beyond a certain distance from the point looked at is an area of blankness as regards perception of form, however clearly comparatively large masses of light and shade may be perceived. We read not by letters but by words, and for easy reading the eye should be able when absolutely fixed to see at least a whole word with fair distinctness, and have a fair inkling of the next. A moderately accurate idea of more words than this is, of course, often gained, but with a long line the dilemma is much increased by the beginning of the line passing away into the area of form-blindness before the end is reached, so that the back spring becomes a "leap in the dark."

In all cases, however, there is no doubt that the finding of the next line is much facilitated by type being "leaded," so that there is a continuous white line between each row of letters, along which the eye can unconsciously trace its way back, and the same may be said of pretty frequent "breaks" in the uniformity of the page by breaking the matter into paragraphs, each beginning with an indent.

From what we have said it is obvious that the length of line should vary according to the size of the type. Now, what is the best size of type? According to Messrs. Griffling and Franz, no type smaller than eleven point should be used, this size corresponding about to the long primer in which our leading articles are printed. Most readers, however, would be content with a little less than this, so long as the line is short and the spacing good. The leaders in the *Times* and the whole of the *Lancet* and *British Medical Journal* are printed in smaller type, but there is a considerable difference in the facility with which they can be read. The *British Medical Journal* has far the longest lines, ours and those of the *Lancet* are the same, while the *Times* has the shortest of all; and moderate shortness of line certainly makes for ease of reading so far as eyesight is concerned.

